# Computed Tomography-Based Radiomics for Preoperative Prediction of Tumor Deposits in Rectal Cancer

**DOI:** 10.3389/fonc.2021.710248

**Published:** 2021-09-27

**Authors:** Yumei Jin, Mou Li, Yali Zhao, Chencui Huang, Siyun Liu, Shengmei Liu, Min Wu, Bin Song

**Affiliations:** ^1^ Department of Radiology, West China Hospital of Sichuan University, Chengdu, China; ^2^ Department of MRI, Qujing First People’s Hospital, Qujing, China; ^3^ Department of Research Collaboration, R&D Center, Beijing Deepwise & League of PHD Technology Co., Ltd, Beijing, China; ^4^ Pharmaceutical Diagnostics, GE Healthcare, Beijing, China; ^5^ Huaxi MR Research Center (HMRRC), Department of Radiology, West China Hospital of Sichuan University, Chengdu, China

**Keywords:** tumor deposits, rectal cancer, radiomics, computed tomography, preoperative prediction

## Abstract

**Objective:**

To develop and validate a computed tomography (CT)-based radiomics model for predicting tumor deposits (TDs) preoperatively in patients with rectal cancer (RC).

**Methods:**

This retrospective study enrolled 254 patients with pathologically confirmed RC between December 2017 and December 2019. Patients were divided into a training set (n = 203) and a validation set (n = 51). A large number of radiomics features were extracted from the portal venous phase images of CT. After selecting features with L1-based method, we established Rad-score by using the logistic regression analysis. Furthermore, a combined model incorporating Rad-score and clinical factors was developed and visualized as the nomogram. The models were evaluated by the receiver operating characteristic curve (ROC) analysis and area under the ROC curve (AUC).

**Results:**

One hundred and seventeen of 254 patients were eventually found to be TDs^+^. Rad-score and clinical factors including carbohydrate antigen (CA) 19-9, CT-reported T stage (cT), and CT-reported peritumoral nodules (+/-) were significantly different between the TDs^+^ and TDs^-^ groups (all *P* < 0.001). These factors were all included in the combined model by the logistic regression analysis (odds ratio = 2.378 for Rad-score, 2.253 for CA19-9, 2.281 for cT, and 4.485 for peritumoral nodules). This model showed good performance to predict TDs in the training and validation cohorts (AUC = 0.830 and 0.832, respectively). Furthermore, the combined model outperformed the clinical model incorporating CA19-9, cT, and peritumoral nodules (+/-) in both training and validation cohorts for predicting TDs preoperatively (AUC = 0.773 and 0.718, *P =* 0.008 and 0.039).

**Conclusions:**

The combined model incorporating Rad-score and clinical factors could provide a preoperative prediction of TDs and help clinicians guide individualized treatment for RC patients.

## Introduction

Rectal cancer (RC) is one of the most common cancers and a leading cause of cancer-related death worldwide (1, 2). Tumor deposits (TDs) in RC have been shown to be an important marker of poor prognosis (3–5). This adverse association persists even in those patients with lymph node metastasis (LNM), strongly suggesting that their effect on prognosis is separate and additive (3). Detecting TDs in advance is very important for assessing prognosis of RC patients.

TDs, also called extranodal TDs, peritumoral deposits, or satellite nodules, are defined as discrete tumor foci in the pericolic or perirectal fat, without histological evidence of residual lymph node or identifiable vascular or neural structures (6, 7). According to the eighth edition of the American Joint Committee on Cancer (AJCC) TNM staging system, any T lesions with negative regional LNM and positive TDs are classified as N1c (8). Positive TDs can elevate clinical stages of RC patients. For example, a stage I patient (T1-2N0) with TDs should be reclassified and treated as stage III (T1-2N1c). The early identification of TDs is important for evaluating the stage and treatment plan.

Rectal magnetic resonance imaging (MRI), computed tomography (CT), and endorectal ultrasound are the first-line examinations in RC. However, no imaging modality has been proved to be reliable to predict TDs (9–12). Currently, the diagnosis of TDs still depends on the pathology after surgery, which is not conducive to the early evaluation of tumor characteristics (9). In recent years, radiomics has attained ability of processing medical images and understanding information invisible to human eyes, and it has been widely used in tumor research. Chen et al. (11) and Yang et al. (12) established radiomics models based on ultrasound or MRI for predicting TDs. However, the sample sizes in these studies were small (TDs^+^: 23-40). At present, there is still a lack of CT-based radiomics research in this field. Therefore, we aimed to evaluate predictive value of CT-based radiomics for TDs prediction in a bigger cohort of RC patients.

## Materials and Methods

### Patients

This study was approved by the local Institutional Review Boards (No. 2019-1159, Date: 2019/12/26), and the need for written informed consent was waived.

**Figure 1:**
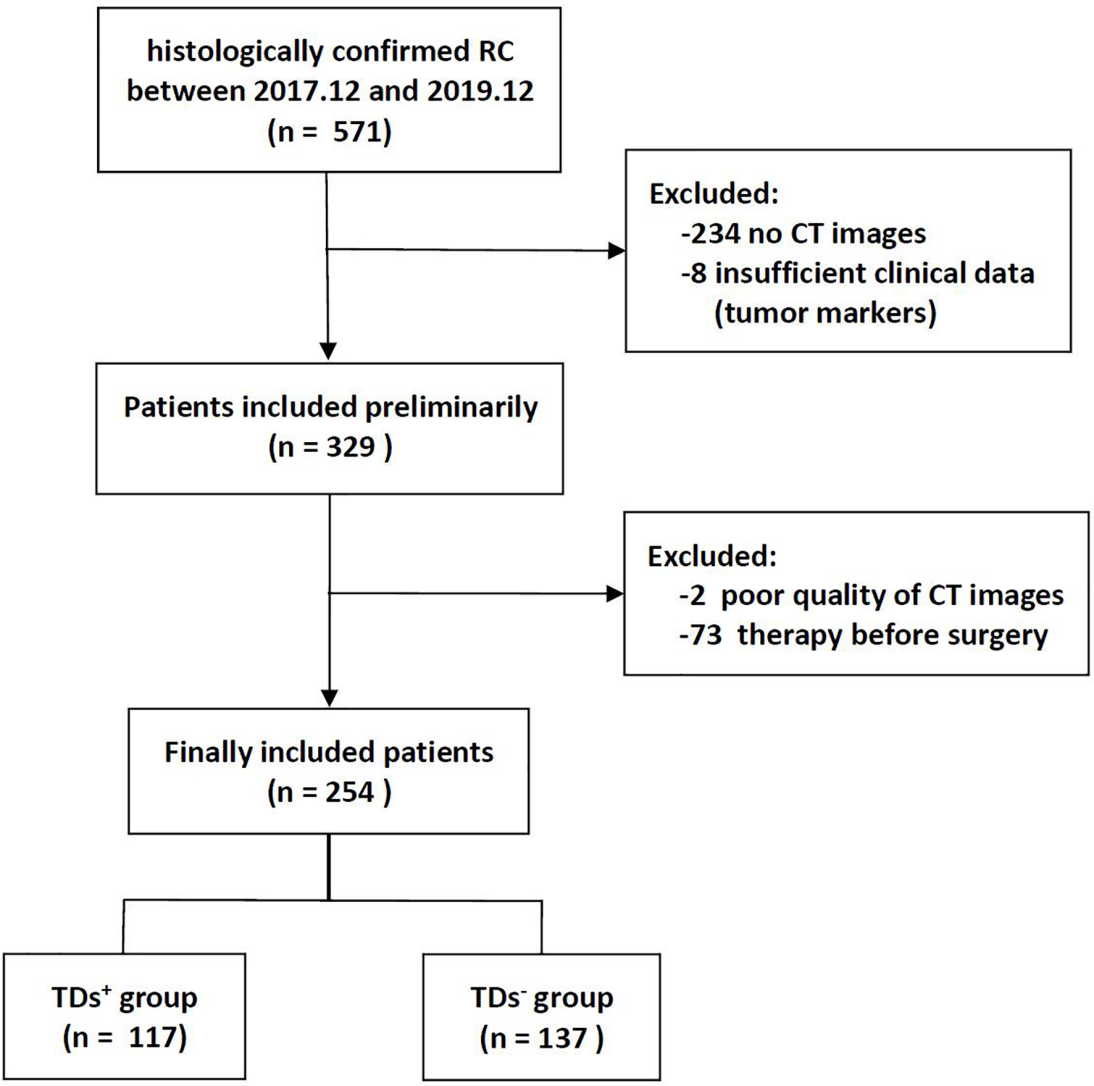
Flowchart of patients’ recruitment pathway.

The institutional database of medical records was searched for suitable patients between December 2017 and December 2019. A total of 254 patients with pathologically confirmed RC (mean age 59.2 years, age range 32-86 years) were finally enrolled according to the following criteria. The inclusion criteria: (1) Patients with pathologically confirmed RC; (2) Sufficient clinical data [e.g., carcinoembryonic antigen (CEA), carbohydrate antigen (CA) 19-9, and CA125]; (3) no prior therapy before surgery. The exclusion criteria: (1) CT scanning was not performed (n = 234); (2) Image quality was poor (n = 2); (3) Lack of tumor markers (n = 8); (4) Patients with other malignant tumors besides RC; (5) Patients receiving neoadjuvant chemoradiotherapy (nCRT) (n = 73). The flowchart of patient recruitment is shown in **Figure 1**. The baseline characteristics and pathological data of patients are listed in **Table 1**. The patients were divided into two groups, namely the training set (n = 203) and the validation set (n = 51), at a ratio of 8:2 according to the scanning date.

**Table 1:** Baseline characteristics of the study population.

Characteristics	TD positive (n = 117)	TD negative (n = 137)	*P* value	training cohort (n = 203)	validation cohort (n = 51)	*P* value
Age (mean ± SD,years)	59 ± 13	60 ± 11	0.553	59 ± 11	59 ± 14	0.682
Gender (men/women)	62/55	89/48	0.056	112/91	39/12	**0.006**
Volume (median,cm^3^)	15.1	12.0	**0.042**	14.0	13.0	0.829
Location			0.078			0.157
upper	61	56		89	28	
middle-lower	56	81		114	23	
cT stage (T1-2/T3/T4)	11/84/22	57/70/10	**<0.001**	53/127/23	15/27/9	0.753
Peritumoral nodules (+/-)^1^	103/14	73/64	**<0.001**	140/63	36/15	0.822
CEA (+/-)	61/56	48/89	**0.006**	87/116	22/29	0.971
CA19-9 (+/-)	44/73	22/115	**<0.001**	50/153	16/35	0.326
CA125 (+/-)	11/106	6/131	0.110	13/190	4/47	0.713
Rad-score	0.60 ± 0.19	0.42 ± 0.20	**<0.001**	0.49 ± 0.21	0.53 ± 0.21	0.343
pT stage (T1/2/3/4)	0/7/91/19	7/37/86/7	**<0.001**	6/34/145/18	1/10/32/8	0.533
pN stage (N0/1/2)	0/69/48	80/43/14	**<0.001**	64/90/49	16/22/13	0.897
Histologic grade (1/2/3)	0/86/31	3/114/20	**0.008**	1/158/44	2/42/7	0.085

^1^Peritumoral nodule was defined as any nodule (diameter > 3mm) within the lymphatic drainage space of rectal cancer on CT images. P values less than 0.05 are shown in bold.

### CT Examination

In our hospital, the chest-abdomen-pelvis contrast-enhanced CT is routinely used in patients with clinically suspected RC for evaluating the primary tumor and metastasis. In this study, CT scanning was performed on a 128-MDCT scanner (Somatom Definition AS+, Siemens Healthcare Sector, Forchheim, Germany) and a dual-source CT system (Somatom Definition Flash, Siemens Healthcare Sector, Forchheim, Germany). Both CT scanners used the same main parameters, as shown in **Supplementary Material**. The radiomics features were extracted from the portal venous phase images.

### Reference Standard for Pathology

TDs were pathologically proven based on surgical specimens. Pathological confirmatory reports were acquired from medical records of the Department of Pathology. The numbers of LN and TDs were calculated and reported in the pathological reports.

### CT Evaluation

Two experienced radiologists (10 and 5 years’ experience in the diagnosis of RC) were assigned to review CT images, without any patient identification and clinicopathological information. Because of limited ability of CT to distinguish T1 from T2 lesions, T1 and T2 lesions were classified as one group (T1-2 group). The nodules with diameter > 3 mm within the lymphatic drainage space of RC on CT images were defined as peritumoral nodules. The inter-observer reliability of CT-reported T stage (cT) and peritumoral nodules (+/-) was evaluated by a weighted kappa statistics test. Then any disagreement between the two readers was solved by discussion during the image interpretation. The results of cT and peritumoral nodules (+/-) are shown in **Table 1**.

### Feature Extraction and Model Building

**Figure 2:**
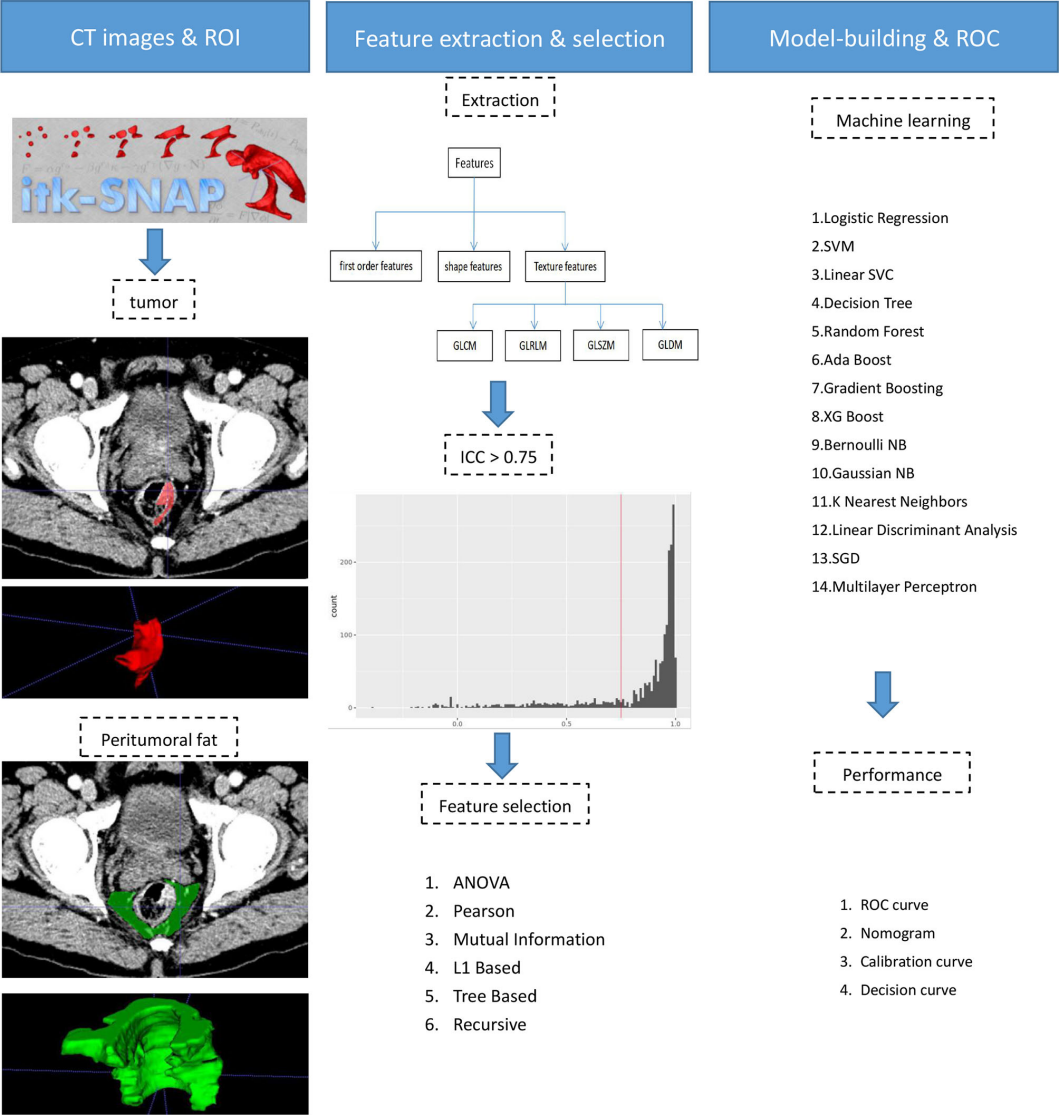
Radiomics workflow.

The tumoral and peritumoral regions in all patients were separately drawn slice by slice to obtain intra- and peritumoral features (**Figure 2**). The radiologists selected 20 patients randomly for evaluating feature stability. For the intra-class correlation analysis, one radiologist drew volumes of interest (VOI) twice (one month apart). The inter-observer correlation coefficient was calculated by comparing VOIs of radiologist 1 (first time) and radiologist 2. It is commonly admitted that intra- and inter-class correlation coefficient (ICC) < 0.5 indicates poor reliability, 0.5 - 0.75: moderate reliability, and > 0.75: good or excellent reliability (13). Thus, the features with ICC ≤ 0.75 were excluded.

The CT images were resampled to a pixel spacing of 1.0 mm in three anatomical directions. High-pass and low-pass wavelet filters, Laplacian of Gaussian (LoG) filters with different σ parameters, and the other image transformation methods such as square, square root, logarithm, exponential, gradient, lbp2d, and lbp3 were employed to pre-process original images. Then, we extracted radiomics features (i.e., the first-order, shape, and texture features) by using PyRadiomics (14). The texture features included the following types: the gray-level co-occurrence matrix (GLCM), the gray-level run-length matrix (GLRLM), the gray-level size zone matrix (GLSZM), and the gray-level dependence matrix (GLDM). Finally, a total of 2107 features were extracted from original and filtered images. To eliminate the differences in the value scales, all features were normalized by the z-score analysis. Redundant features were randomly removed by correlation analysis with a threshold of 0.5. Then different feature-selection and machine-learning methods were combined to form 84 classifiers, as shown in **Supplementary Material**. The optimal parameters of radiomics were adjusted to output the best classifier (Rad-score).

The Rad-score and clinical factors were assessed by the univariate logistic regression analysis. The features revealed as statistically significant were then involved into the multivariate logistic regression analysis for constructing the combined model. A nomogram was generated for the model visualization, graphical evaluation of variable importance, and the calculation of predictive accuracy. The Hosmer-Lemeshow test was performed to assess the goodness-of-fit of the nomogram. A calibration curve, obtained by plotting the actual TDs^+^ probability against the nomogram-predicted probability of TDs^+^, was used to assess the calibration of the nomogram (15). Decision curve first introduced in 2006 by Vickers et al. (16) was used to evaluate clinical utility of the nomogram. The receiver operating characteristic curve (ROC) analysis was performed to assess the predictive performance of the models.

### Statistical Analysis

Student’s *t* test, non-parametric test, chi-squared test, and Fisher’s exact test (where appropriate) were used to analyze differences of baseline characteristics in **Table 1**. The area under the ROC curve (AUC) was compared by Delong’s test. The software used in this study included SPSS 21.0 software (IBM), Python 3.6, Stata 15.0, and Medcalc 15.2.2. The confidence level was set at *P* < 0.05.

## Results

### Patients’ Characteristics

A total of 254 patients were enrolled in this study, in which 117 patients were TDs^+^. As shown in **Table 1**, there were significant differences in volume (*P* = 0.042), cT (*P* < 0.001), peritumoral nodules (+/-) (*P* < 0.001), CEA (*P* = 0.006), CA19-9 (*P* < 0.001), and pathological factors [i.e., T stage (*P* < 0.001), N stage (*P* < 0.001), and grade (*P* = 0.008)] between the TDs^+^ and TDs^-^ groups. Between the training and validation cohorts, there was a significant difference in gender. The weighted kappa coefficients of cT and peritumoral nodules (+/-) between two radiologists were 0.656 [95% confidence interval (CI): 0.570-0.741] and 0.679 (95%CI: 0.584-0.774) in the whole cohort, which showed substantial consistency according to the generally accepted knowledge: 0.41-0.60, moderate, 0.61-0.80, substantial, and 0.81-1.00, almost perfect (17).

### Feature Selection and Model Building

For the consistency test of VOIs, 1490 tumoral and 1605 peritumoral features had good reliability with ICC > 0.75. Rad-score involving 10 peritumoral and 3 tumoral features was finally established by the logistic regression analysis. The 13 features and their coefficients are shown in **Supplementary Material**. Rad-score had statistical difference between the TDs^+^ and TD^-^ groups (0.60 ± 0.19 *vs* 0.42 ± 0.20, *P* < 0.001).

A clinical model was composed of three factors selected by the logistic regression analysis, namely CA19-9, cT, and peritumoral nodules (+/-). The combined model was built by adding Rad-score to the clinical model [odds ratio (OR) = 2.378 for Rad-score, 2.281 for cT, 4.485 for peritumoral nodules (+/-), and 2.253 for CA19-9], as summarized in **Table 2**. Although volume and CEA were significantly different between the TDs^+^ and TDs^-^ groups, they were both excluded by the multivariate logistic regression analysis (**Table 2**).

**Table 2:** Risk factors selected by the logistic regression analysis.

Variables	Univariate		Multivariate	
	OR	*P-*value	OR	*P-*value^1^
Age	0.996	0.745		
Gender	0.624	0.098		
Volume	1.000	0.918		
Location	2.216	**0.006**	0.677	0.267
cT stage	3.496	**<0.001**	2.281	**0.009**
Peritumoral nodules (+/-)	6.009	**<0.001**	4.485	**<0.001**
CEA	1.725	0.057		
CA19-9	2.928	**0.002**	2.253	**0.045**
CA125	2.779	0.098		
Rad-score	2.771	**<0.001**	2.378	**<0.001**

^1^If P > 0.1, variables were excluded from the combined model. P values less than 0.05 are shown in bold.

**Figure 3:**
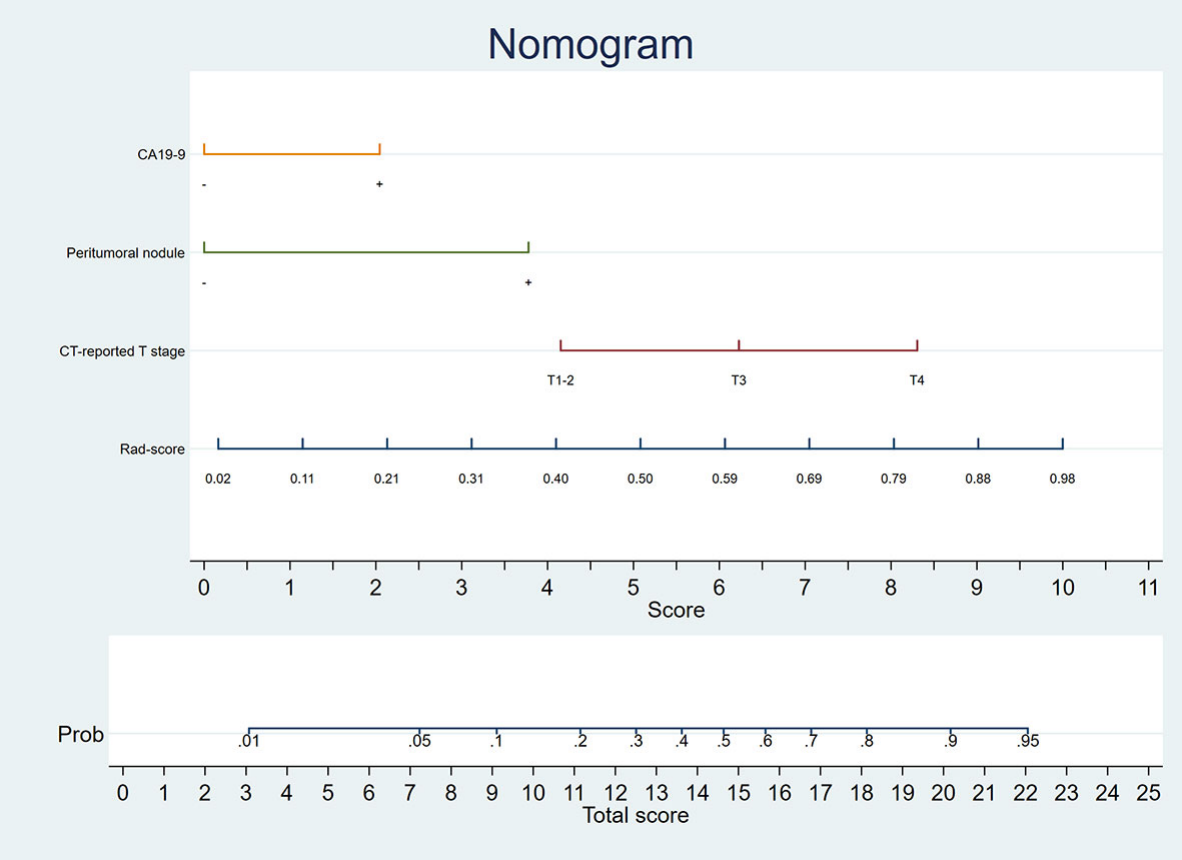
Nomogram developed in the training cohort.

**Figure 4:**
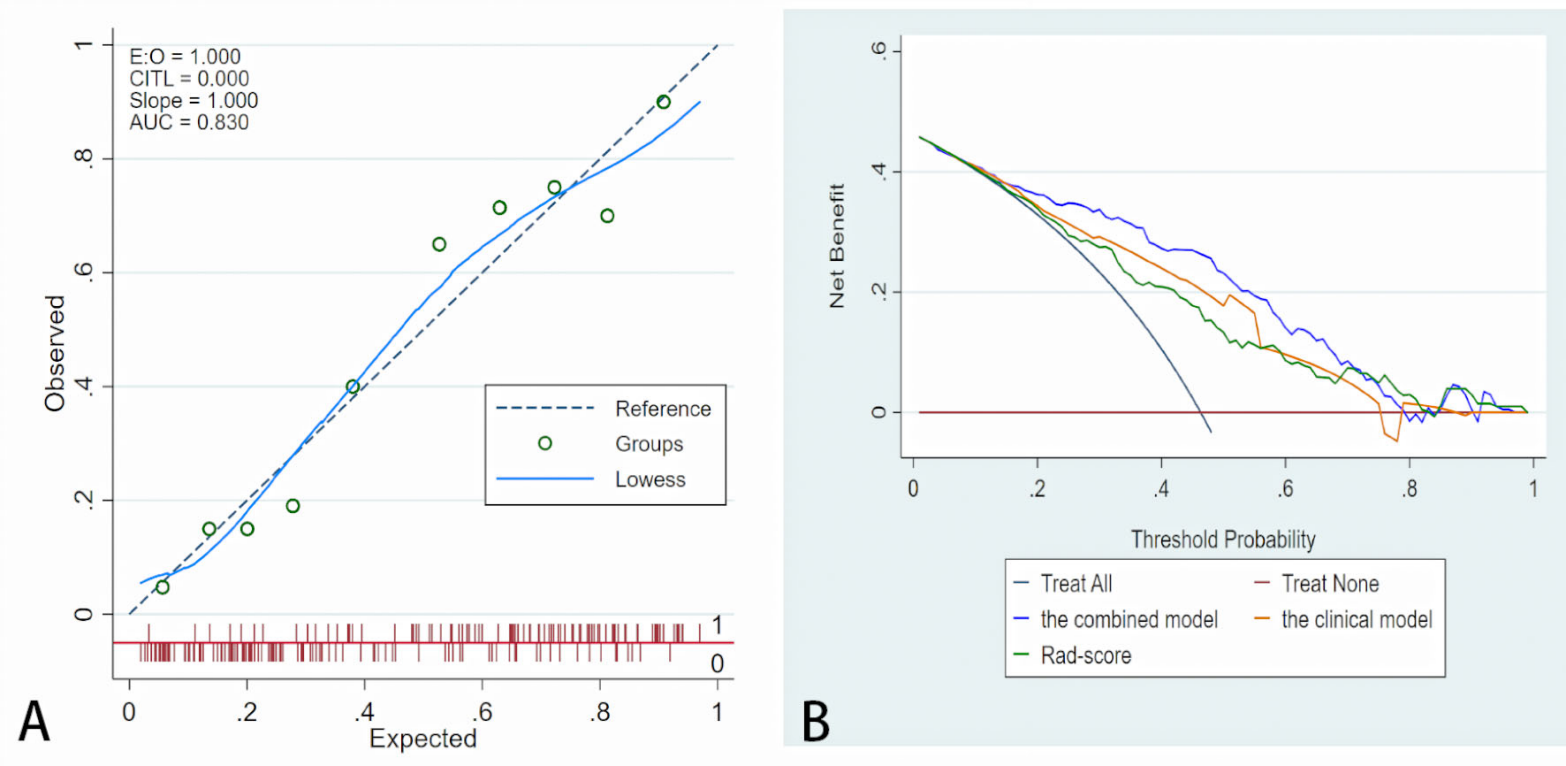
Fit and usefulness evaluation of the nomogram. **(A)** Calibration curve of the nomogram. The calibration curve depicts the calibration of the model in terms of the agreement between the predicted risk of TDs (x axis) and observed outcomes of TDs (y axis). The blue solid line represents the performance of the nomogram (Note: a closer fit to the diagonal dotted line represents a better prediction). **(B)** The decision curve demonstrates that the model obtains more benefit than “treat all”, “treat none”, Rad-score, and the clinical model, when the threshold probability is in the range of 18% to 70%.

A nomogram was generated for visualizing the combined model (**Figure 3**). In the nomogram, the point for each variable on the corresponding axis can be added to determine the risk of TDs^+^. Higher total score was associated with greater risk of TDs^+^. The combined model had a good fit according to the Hosmer-Lemeshow test (*P* = 0.642 > 0.05). The calibration curve of the nomogram demonstrated a good agreement between the predicted probability and actual observed probability (**Figure 4A**), because the solid line was close to the reference line (dotted line). However, this model underestimated actual risk of TDs^+^ (the range of the threshold probability: 30%-75%) and overestimated risk when threshold probability > 75%. The decision curve was performed to assess clinical usefulness of the combined model (**Figure 4B**), showing that the combined model obtained more benefit than “treat all”, “treat none”, Rad-score, and the clinical model, when the threshold probability was between 18% and 70%.

### Model Comparisons

**Figure 5:**
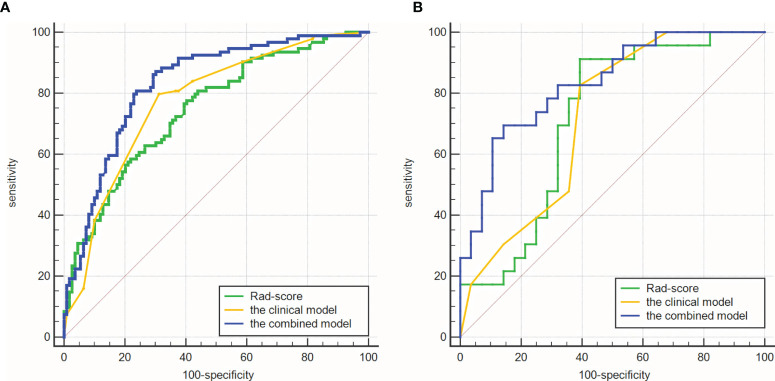
Comparisons of ROC curves. **(A)** in the training cohort. **(B)** in the validation cohort. The combined models had higher AUCs (0.830 and 0.832) than the clinical model (0.773 and 0.718).

The AUCs of the clinical model were 0.773 (95%CI: 0.709-0.829) in the training cohort and 0.718 (95%CI: 0.575-0.835) in the validation cohort. Rad-score had similar AUCs with the clinical model (0.747, 95%CI: 0.681-0.805 and 0.717, 95%CI: 0.574-0.835). Improved predictive value was achieved by adding Rad-score to the clinical model. In detail, the AUCs of the combined model were higher than those of the clinical model in the training and validation cohorts (0.830 and 0.832; *P* = 0.008 and 0.039). As shown in **Table 3** and **Figure 5**.

**Table 3:** ROC analyses of the models in the training and validation cohorts.

Model	The training cohort			*P* value	The validation cohort			*P* value
	AUC	SEN (%)	SPE (%)		AUC	SEN	SPE	
Rad-score	0.747 (95%CI: 0.681-0.805)	77.7	59.6	**0.001**	0.717 (95%CI: 0.574-0.835)	91.3	60.7	0.054
Clinical model	0.773 (95%CI: 0.709-0.829)	80.9	63.3	**0.008**	0.718 (95%CI: 0.575-0.835)	82.6	60.7	**0.039**
Combined model	0.830 (95%CI: 0.771-0.879)	80.9	76.2		0.832 (95%CI: 0.701-0.922)	78.3	71.4	

P values: compared with the combined model. P values less than 0.05 are shown in bold.

### Subgroup Analyses

The results of subgroup analyses were listed in **Table 4**. In our study, there were 35 patients with N1c in the TDs^+^ group. The values of the combined model were significantly different between the N1c group and the rest TDs^+^ (n = 82) (0.55 ± 0.27 *vs* 0.69 ± 0.19, *P* = 0.002). However, the clinical model had no significant difference between them (*P* = 0.113). For differentiating N1c from TDs^-^ patients, the combined model had an AUC of 0.741 (95%CI: 0.669-0.805), which was not significantly higher than that of the clinical model (0.711, 95%CI: 0.637-0.778; *P* = 0.326). In the rest TDs^+^ group, the combined model outperformed the clinical model in identifying TDs^+^ from TDs^-^ patients (AUC = 0.864 *vs* 0.781, *P* < 0.001).

**Table 4:** Subgroup analyses of the models in the whole cohort.

Subgroups	The clinical model				The combined model				*P*
	value	AUC	SEN	SPE	value	AUC	SEN	SPE	
TD+									
N1c (n = 35)	0.56 ± 0.21	0.711 (95%CI: 0.637-0.778)	74.3%	67.2%	0.55 ± 0.27	0.741 (95%CI: 0.669-0.805)	80.0%	59.9%	0.326
TDs^+^ except N1c (n = 82)	0.62 ± 0.18	0.781 (95%CI: 0.721-0.834)	82.9%	67.2%	0.69 ± 0.19	0.864 (95%CI: 0.812-0.907)	87.8%	74.5%	**<0.001**
Number of TDs									
1-2 (n = 77)	0.58 ± 0.21	0.732 (95%CI: 0.668-0.790)	75.3%	67.2%	0.62 ± 0.24	0.800 (95%CI: 0.740-0.852)	84.4%	66.4%	**0.005**
≥3 (n = 40)	0.64 ± 0.16	0.814 (95%CI: 0.749-0.869)	90.0%	67.2%	0.72 ± 0.19	0.880(95%CI: 0.823-0.924)	90.0%	75.9%	**0.005**
Pathological T stage									
T1-2		0.519 (95%CI: 0.375-0.661)	57.1%	52.3%		0.740 (95%CI: 0.598-0.853)	57.1%	97.7%	**0.028**
T3-4		0.732 (95%CI: 0.665-0.791)	86.4%	55.9%		0.789(95%CI: 0.726-0.843)	80.9%	65.6%	**0.033**
Peritumoral nodules on CT									
+ (n = 176)		0.661 (95%CI: 0.586-0.730)	91.3%	38.4%		0.771 (95%CI: 0.701-0.831)	85.4%	57.5%	**0.003**
- (n = 78)		0.667 (95%CI: 0.552-0.770)	85.7%	42.2%		0.751 (95%CI: 0.640-0.842)	57.1%	82.8%	0.263
Clinical stage									
II (n = 49)		0.550 (95%CI: 0.401-0.692)	41.7%	73.0%		0.721 (95%CI: 0.574-0.839)	50.0%	89.2%	0.180
III (n = 176)		0.661 (95%CI: 0.586-0.730)	91.3%	38.4%		0.771 (95%CI: 0.701-0.831)	85.4%	57.5%	**0.003**

P value: comparison between the clinical model and combined model. P values less than 0.05 are shown in bold.

In TDs^+^ group, there were 77 patients with 1-2 TDs and 40 patients with ≥ 3 TDs. The group with ≥ 3 TDs had higher values of both combined and clinical models than the 1-2 TDs group (*P* = 0.015 for combined model, and 0.08 for the clinical model). Moreover, the combined model outperformed the clinical model in both 1-2 and ≥ 3 TDs^+^ groups when differentiating TDs^+^ from TDs^-^ patients (both *P* = 0.005).

The patients with peritumoral nodules on imaging were all classified as clinical stage III in this study. The combined model had moderate diagnostic performance (AUC = 0.771, 95%CI: 0.701-0.831) in the stage III patients. As for patients without peritumoral nodules on imaging, the combined model also showed moderate diagnostic performance with an AUC of 0.751. As for patients with different pathological T stages, the combined model had similar AUCs between the T1-2 and T3-4 groups (0.740 and 0.789).

## Discussion

In this study, a combined model incorporating Rad-score, CA19-9, cT, and peritumoral nodules (+/-) was established based on CT in a bigger cohort (compared with the previous studies), showing potential to predict TDs in RC. This combined model outperformed the clinical model in predicting TDs (AUC = 0.830 *vs* 0.773, *P* = 0.008 in the training cohort; 0.832 *vs* 0.718, *P* = 0.039 in the validation cohort), indicating that adding Rad-score to the clinical factors improved the predictive value.

TDs are an important prognostic factor in RC. A meta-analysis reported that a total of 21 included studies all found a significantly worse prognosis in patients with TDs (3). Goldstein et al. (18) found that when patients with differing numbers of LNM were assessed separately, those with TDs still demonstrated a worse prognosis. For example, with one positive node 5-year survival was 62% with no TDs detected *versus* 44% with TDs. When six or more LNs were involved 5-year survival was 16% without TDs *versus* 3% with TDs. This result strongly suggests that the effect of TDs on prognosis is separate from that of LNM. Thus, preoperative prediction of TDs is of great significance to assess the prognosis of patients with LNM or without LNM (N1c). The selection of treatment strategies mostly depends on cancer staging. According to the eighth edition of the AJCC TNM staging system, the presence of TDs without LNM causes patients to be classified as N1c, and these patients are staged as III. That is, once TDs are present, nCRT is recommended. If TDs status is absent pretherapeutically, the treatment plan may be misguided.

Traditional imaging techniques, such as CT, MRI, and US, that depend on the naked eye cannot reliably assess the condition of TDs. Recently, radiomics has appeared as a potent tool for constructing decision-support models. Researchers have started to use radiomics to predict TDs in RC. Chen et al. (11) developed a ultrasound radiomics model with an AUC of 0.795 in a cohort of 127 patients (TDs^+^: n = 40). Yang et al. (12) established a MRI-based radiomics model in 139 RC patients (TDs^+^: n = 23), which had an AUC of 0.820. Our results showed a comparable AUC with the previous studies in a bigger cohort (254 patients; TDs^+^: n = 117). We included T stage in the combined model, which was consistent with Yang et al. (12). Different from Yang et al. (12) [two-dimensional (2D) region of interest (ROI)], we established the model based on 3D ROI, namely VOI. 2D ROI did not cover the whole lesion, and thus some information of tumor heterogeneity may be lost.

In this study, “peritumoral nodule” was defined as any nodule (diameter > 3 mm) within the lymphatic drainage space of RC, involving LNM and TDs. The CT-reported factors (i.e., cT and peritumoral nodule) were reviewed by two experienced radiologists, and thus reliable data were acquired. The volume in the TDs^+^ group was larger than that of the TDs^-^ group (median: 15.1 *vs* 12.0 cm^3^), which was consistent with the conclusion of Wei et al. (19). Although elevated CEA was found in the TDs^+^ group, CEA was not included in the combined model. Peritumoral features accounted for the majority of features in Rad-score (10/13, 76.9%), suggesting the important role of environment around the rectum in the formation of TDs (20).

Although AJCC has not correlated a higher number of TDs with staging, which is unlike LNs (e.g., N1: 1 to 3 regional LNs, N2: ≥ 4 regional LNs) (8). Several authors have found a significant relationship between an increasing number and worsening of prognosis (18, 21, 22). For example, in patients with ≥ 3 TDs, none was alive at 5-year follow up. It is worthy of note that this is significantly worse than patients who had similar number of LNM (in fact even those with ≥ 6 positive LNs had a 5-year survival of 11%). In our study, the group with ≥ 3 TDs had higher value of the combined model than the 1-2 TDs group (*P* = 0.015), indicating that the combined model was helpful for predicting the number of TDs. Moreover, the N1c group had lower value of the combined model than the rest TDs^+^ group (*P* = 0.002), suggesting possibility of the combined model for predicting N1c. In the future, a large multicenter study is certainly needed to confirm these observations.

The patients with peritumoral nodules on imaging were all classified as clinical stage III in this study. The combined model had moderate diagnostic performance in the stage II and III patients, indicating the good stability of the model. There were 78 patients without peritumoral nodules on imaging, in which 14 patients were TDs^+^. Because of the small sample size, the diagnostic performance of the combined model (AUC = 0.751) was not accurate here. More cases are needed to verify this result.

Our study had several limitations. First, the selection bias existed due to the retrospective design. Second, prospective and external validation was not performed. Third, because it is impossible to achieve one-to-one correspondence between pathological and radiological peritumoral nodules in this study, we delineated the whole peritumoral area in the lymphatic drainage space of RC. Finally, we excluded nodules with diameter < 3 mm on imaging, while there was still a risk of TDs in these small nodules (23).

In conclusion, the CT-based radiomics model is helpful for the preoperative prediction of TDs in RC patients.

## Data Availability Statement

The original contributions presented in the study are included in the article/**Supplementary Material**. Further inquiries can be directed to the corresponding authors.

## Ethics Statement

The studies involving human participants were reviewed and approved by West China Hospital of Sichuan University Biomedical Research Ethics Committee. Written informed consent for participation was not required for this study in accordance with the national legislation and the institutional requirements.

## Author Contributions

BS and MW conceived of the presented idea. YJ and SML collected the data. ML, YZ, and CH analyzed the data. ML drafted the manuscript. SYL improved the quality of English. All authors contributed to the article and approved the submitted version.

## Conflict of Interest

Authors YZ and CH were employed by the company Beijing Deepwise & League of PHD Technology Co., Ltd. Author SYL was employed by the company GE Healthcare (Beijing).

The remaining authors declare that the research was conducted in the absence of any commercial or financial relationships that could be construed as a potential conflict of interest.

## Publisher’s Note

All claims expressed in this article are solely those of the authors and do not necessarily represent those of their affiliated organizations, or those of the publisher, the editors and the reviewers. Any product that may be evaluated in this article, or claim that may be made by its manufacturer, is not guaranteed or endorsed by the publisher.
