# Supplementary material for: Computed Tomography-Based Radiomics for Preoperative Prediction of Tumor Deposits in Rectal Cancer
Source: Front Oncol. 2021 Sep 27;11:710248. doi: 10.3389/fonc.2021.710248 (PMC8502898; doi:10.3389/fonc.2021.710248)
Supplement: Supplementary file 1 [file DataSheet_1.docx]

Supplementary Material

**CT parameters:**

Parameters: voltage, 120 kV; amperage, 200-210 mA; rotation time, 0.5 seconds; pitch, 1.0; and section thickness, 2.0 mm.

An anionic contrast medium (Omnipaque 350; GE Healthcare, Chicago, IL), administered at a dose of 1.5 mL/kg body weight, was injected intravenously using a power injector at a rate of 3 mL/s.

**Feature selection methods:**

1. ANOVA
2. Pearson
3. Mutual Information
4. L1 Based
5. Tree Based
6. Recursive

**The features included in Rad-score:**

(note：those with “_peri” were extracted from the peritumoral area)

| **feature** | **coef** | **mean** | **standard deviation** |
| --- | --- | --- | --- |
| 1.squareroot_gldm_DependenceEntropy_peri | -0.5955 | 7.1014 | 0.2468 |
| 2.wavelet-HLH_glszm_LowGrayLevelZoneEmphasis_peri | 0.3939 | 0.2690 | 0.1660 |
| 3.wavelet-HLH_glszm_GrayLevelNonUniformity_peri | -0.4071 | 18.4576 | 17.0646 |
| 4.wavelet-HHL_glcm_ClusterShade_peri | 0.4487 | 0.0177 | 0.0434 |
| 5.wavelet-LHL_glcm_ClusterShade_peri | -0.1252 | -3.2244 | 11.2140 |
| *6.Log-sigma-3-0-mm-3D_gldm_DependenceVariance* | 0.0587 | 41.9886 | 5.2325 |
| *7.log-sigma-5-0-mm-3D_glcm_ClusterShade* | 0.1579 | 2.2334 | 64.7158 |
| 8.lbp-2D_firstorder_90Percentile_peri | -0.0114 | 8.9803 | 0.1390 |
| 9.lbp-2D_glszm_SizeZoneNonUniformity_peri | 0.1498 | 1.0855 | 0.3687 |
| 10.wavelet-HHH_firstorder_Skewness_peri | 0.0476 | -0.0012 | 0.0439 |
| 11.wavelet-HHL_firstorder_Mean_peri | 0.0332 | -0.0358 | 0.0448 |
| 12.wavelet-LHH_firstorder_Median_peri | 0.0796 | -0.0348 | 0.0480 |
| *13.lbp-2D_glcm_SumEntropy* | -0.0136 | -0.0000 | 0.0000 |

**Machine learning:**

1. Logistic Regression
2. SVM
3. Linear SVC
4. Decision Tree
5. Random Forest
6. Ada Boost
7. Gradient Boosting
8. XG Boost
9. Bernoulli NB
10. Gaussian NB
11. K Nearest Neighbors
12. Linear Discriminant Analysis
13. SGD
14. Multilayer Perceptron

**The formula of the combined model**

=1/(1+EXP(-(-5.815+0.812×CA19-9+0.825×cT+1.501×peritumoral nodules (+/-)+4.051×Rad-score)))
